# Prevalence and outcome of stress hyperglycaemia among severely malnourished children admitted to Mulago referral and teaching hospital in Kampala, Uganda

**DOI:** 10.1186/s40795-018-0258-3

**Published:** 2018-12-12

**Authors:** Anita Tumwebaze, Elizabeth Kiboneka, Jamir Mugalu, Edward M. Kikabi, James K. Tumwine

**Affiliations:** Department of Paediatrics and Child Health, School of Medicine, College of Health Sciences, Makerere University, Kampala, Uganda

**Keywords:** Stress hyperglycaemia, Severe acute malnutrition

## Abstract

**Background:**

Stress hyperglycaemia is a transient increase in blood glucose level during stressful events and is common in critically ill children. Several studies have demonstrated increased risk of mortality in these children. There is paucity of information on this subject in sub Saharan Africa.

The aim of this study was to describe the prevalence, outcome and factors associated with stress hyperglycaemia among children with severe acute malnutrition (SAM) admitted to the Mwanamugimu nutrition unit of Mulago hospital in Uganda.

**Methods:**

This study was conducted from August 2015 to March 2016 at the Mwanamugimu nutrition unit of Mulago hospital among severely malnourished children aged 1 to 60 months. Random blood sugar levels were measured. Stress hyperglycaemia was considered as a random blood sugar > 150 mg/dl. The final outcome was ascertained at death or discharge. Statistical analysis was done using the Chi square test and logistic regression.

**Results:**

Two hundred and thirty-five children were enrolled of whom 50% were girls. The median age was 5.1 months (range 1–60 months). Stress hyperglycaemia was present in 16.6% of the 235 participants. Several factors were significantly associated with stress hyperglycaemia at bivariate analysis; but on logistic regression, only presence of oral sores was associated with stress hyperglycaemia: (Odds ratio 2.61; 95% CI 1.02–6.65).

Mortality was higher among children with stress hyperglycaemia (56.4%) compared to (12.8%) in the non-hyperglycaemic group: OR 8.75; 95% CI 4.09–18.70).

**Conclusion:**

The prevalence of stress hyperglycaemia was 16.6% and was associated with high mortality. It is important to monitor blood glucose levels of severely malnourished children. Hitherto, the main concern among severely malnourished children has been hypoglycaemia. Innovative ways of preventing and managing stress hyperglycaemia among these children are urgently needed.

## Background

Severe acute malnutrition affects an estimated 19 million children under five years of age worldwide and is estimated to account for 400,000 child deaths each year. In Uganda, the prevalence of malnutrition remains high [1]. Children with severe malnutrition have altered glucose metabolism and this usually results into impaired glucose production, clearance and absorption. Impaired insulin response has also been reported in these children [2–4]. The literature suggests that impaired glucose absorption in severe malnutrition leads to oxidative stress [2–4]. Presence of oxidative stress, impaired glucose absorption and infections can lead to the development of stress hyperglycaemia [5]. The management guidelines of children with severe malnutrition usually focus on hypoglycaemia which is a well-known complication of severe malnutrition and not much attention has been given to stress hyperglycaemia [6–8]. These children receive high energy feeds at stipulated intervals (2–3 hourly) during nutritional rehabilitation and at the point of admission, intravenous fluids containing 10% dextrose are usually used in resuscitation [6, 7]. These can potentiate the development of stress hyperglycaemia in severely malnourished children who are usually critically ill [9].

Currently, there is no consensus on the management of stress hyperglycaemia among these children [10, 11]. Stress hyperglycaemia has been documented in critically ill children and occurs even in those with previously normal glucose homeostasis [10, 12–14]. It is defined as a transient increase in blood glucose level usually above the normal physiological post-prandial upper limit for non-diabetic children (> 140 mg/dl) [14]. It has been estimated that about 49% of critically ill children present with stress hyperglycaemia with blood sugar levels above 150 mg/dl but this usually resolves spontaneously when the stressing event is removed [13]. In the acute phase of illness, it is an adaptive mechanism to improve chances of survival but as it persists longer with stressful events, it is associated with increased risk of mortality [13]. Higher levels of hyperglycaemia (RBS > 180 mg/dl) are associated with a higher risk mortality [15]. It has been associated with a number of conditions in children such as hyperthermia, septicaemia or septic shock and hypothermia. All these conditions occur in children with severe acute malnutrition and lead to increased metabolic demand thus putting the body through a period of stress [14]. Stress hyperglycaemia usually resolves with improvement in the clinical condition of the patient and no further testing is usually required [14]. Previous studies have found no association between stress hyperglycaemia and later development of diabetes mellitus [8, 13, 16, 17]. In this study, we determined the prevalence of stress hyperglycaemia among children with severe acute malnutrition and its impact on their outcome during admission in a resource limited setting in Africa. There is paucity of data about stress hyperglycaemia in our setting. Findings from this study will help to contribute to policies on the management of children with SAM in our setting. The main objective of the study was to determine the prevalence and factors associated with stress hyperglycaemia among severely malnourished children admitted to the Mwanamugimu nutrition unit (MNU) of Mulago Hospital in Kampala, Uganda.

We also sought to determine the short-term outcome of children with stress hyperglycaemia in the same unit.

## Methods

This was a cross-sectional study with a follow up component carried out from August 2015 to March 2016.

The study was conducted at the MNU of Mulago National Referral Hospital in Kampala, Uganda. Mulago hospital has a 1500 bed capacity and comprises of two main divisions; − New Mulago and old Mulago. MNU is found in old Mulago where the main paediatric wards are located. MNU is the nutrition ward in Mulago national referral hospital and receives patients from all over the country. It admits all children older than 6 months with severe acute malnutrition who have associated complications and infants less than 6 months who have features of severe acute malnutrition (nutritional oedema or Z scores < -3SD) including those with ineffective breastfeeding, medical complications or other social issues that compromise the nutrition of the infant. The children are admitted through the emergency paediatric unit (Acute Care Unit) where they receive the initial care in the first 24 h. Later, those with severe acute malnutrition are transferred to MNU. Most come from the central region while the rest are referred from hospitals and health centres upcountry. The MNU has 80 beds and on average it admits about 120 patients per month. The unit has 5 paediatricians and 1 medical Officer. There are 17 nurses and, of these, 4 are enrolled nurses, 9 are nursing officers and 4 are nursing assistants. There are 3 nutritionists attached to the unit.

Children on the ward receive feeds at stipulated intervals. In the first 48 to 72 h (critical phase), they receive feeds (F75 for those > 6 months with severe acute malnutrition) and specially diluted therapeutic milk (SDTM) for infants < 6 months with severe acute malnutrition who are not breast feeding adequately) 2 hourly. When children are clinically stable, an appetite test (for those > 6 months) is done to identify children to transit to “Ready to Use Therapeutic Feeds” (RUTF). Specially Diluted Therapeutic Milk (SDTM) is made from F100 and contains 69Kcal/100 ml. It is given to infants less than 6 months who have severe acute malnutrition and are unable to get sufficient breast milk. The critical phase usually lasts 7 to 10 days. Children who pass the appetite test are then transferred to the rehabilitation phase for a few days where the care takers are also taught how to prepare nutritionally rich food for use at home. Children who are stable and feeding well are then transferred to the Outpatient therapeutic Clinic for outpatient management which has been demonstrated to be a better environment for rehabilitation of these children.

### Inclusion criteria

We included children aged 1–60 months admitted to MNU for SAM and who had been on the ward for less than 3 days.

### Exclusion criteria

We excluded children who had no care givers to give information and children with a known or suspected diagnosis of diabetes mellitus.

### Sample size calculation

The sample size was calculated using Kish Leslie formula for prevalence studies [18].

N = ZP (1-P)/D^2^- where N is the required sample size, Z is the value corresponding to 95% confidence interval, D is the absolute error equal to 0.05 and P is the prevalence of hyperglycaemia among children with SAM [14] equal to 11%.

A sample size of 165 was obtained for the first objective assuming 10% for incomplete data.

**The sample size for factors associated with hyperglycaemia** was calculated using the formula for proportions. **N** = (**Z**_**α**/**2**_ + **Z**_**β**_)^**2**^ ∗ (**p**_**1**_(**1** − **p**_**1**_) + **p**_**2**_(**1** − **p**_**2**_))/(**p**_**1**_ − **p**_**2**_)^**2**^**.**

Where N = the required sample size, P1- the prevalence of stress hyperglycemia among children with hyperthermia (a factor associated with hyperglycemia) was assumed to be 9.3%and P2- prevalence among those without hyperglycemia was 2.8% [17]. Hence, a sample size of 208 was attained. Since this was a bigger sample size, it was used for the study.

### Study procedure

The study had 2 research assistants a nurse and a medical officer and one of us (AT). The nurse was responsible for screening the children and obtaining informed written consent from the care givers and also doing the random blood sugar tests and Rapid diagnostic test for malaria. The medical officer carried out physical examination, anthropometry, collected the blood samples and delivered them to the laboratories. AT was in charge of the whole process and was involved in patient recruitment and care.

All children admitted to MNU were screened for eligibility every morning between 8 and 10 am. Informed oral and written consent was obtained from the care givers of the eligible children. A random blood sugar was done using a finger prick at least 2 h from the previous therapeutic feed. The study assistant was always on the ward before 8 am to screen the patients for eligibility and be able to do the random blood sugar measurements just the children received the 8 am feeds. The study assistant also took note of whether the child had received intravenous dextrose and how long they had received it before doing the random blood glucose level. From the data collected, all the children who received 10% dextrose had received it more than 12 h prior to blood testing. Children with hyperglycaemia were referred to the attending team on the ward for follow up. An interviewer administered questionnaire was used to take the history from care takers about the patient such as the demographic information, clinical symptoms and information about the care takers. Patients’ charts were reviewed for treatment received such as antibiotics and intravenous fluids/ dextrose. The final outcome was obtained by reviewing the patient charts on death or discharge from the ward.

#### Examination

Physical examination was done for all the enrolled children and this involved doing anthropometry, general and systemic examination.

Anthropometry was done by taking the weights of the children using a digital scale. The height was measured using a stadiometer for children above 24 months who could stand. For the younger children less than 24 months who could not stand, length was measured using the stadiometer with a child in supine position and stabilised in a straight position with the help of the care taker. The length was reported to the nearest 0.1 cm. Children who were more than 24 months but could not stand had their length measured and an adjustment of 0.7 cm added to the length so as to get an equivalent of their height if they were able to stand. [7] Their nutritional status was determined using the WHO Z score charts taking into the count the presence or absence of nutritional oedema. Children were considered to be SAM if their Weight/height Z score was ≤ -3SD or had nutritional oedema or both.

A systemic physical examination was carried for clinical signs pointing to any co-morbid conditions such as presence of pallor, jaundice, respiratory distress, tachycardia, signs of shock, lethargy, alertness and body temperature was measured. The findings were summarised in the questionnaire.

### Collection and analysis of blood samples

An ‘On Call Plus’ glucometer with matching test strips; an international brand from the ACON Laboratories in the United States of America was used for the blood sugar tests. The glucometer was calibrated using a control solution every after 20 tests or whenever a different batch of test strips was used.

The random blood sugar level was done using a finger prick. The left index finger was used for the test. The side of the finger was swabbed with sterile alcohol swabs containing 70% alcohol and a prick was made with a sterile lancet then a drop of blood was put onto the test strip that was already mounted into the glucometer. A reading of the blood sugar was then obtained after 30 s and recorded in mg/dl. The same finger prick used for random blood sugar was also used to obtain 2 drops of blood to do the RDT.

A blood sample for the other tests was collected from the children using peripheral veins.. The area was first swabbed with sterile cotton swabs containing 70% alcohol and then allowed to air dry for about 60 s. A tourniquet was then applied for a brief period of about 2 min then a sterile autodestruct syringe was used to collect about 5mls of blood. One millilitre(ml) was put into an EDTA container for CBC, 2mls in a general container for serum albumin, urea, creatinine and electrolytes, 2mls was used for blood culture. For all children below 18 months of age, the HIV serology of the mother was done by taking off 1 ml of blood from the mother, if the results came back negative; the child was considered HIV negative. If the mother’s test came back positive, 1.5mls of blood was taken off from the child and sent to Baylor-Paediatric Infectious Diseases Clinic (PIDC) for HIV DNA-PCR to get the HIV status of the child. For children above 18 months of age, HIV serology was using the rapid testing kits (Determine and Stat pak). Children or care givers who were HIV positive were connected to Baylor-PIDC for specialised care. The samples for CBC, electrolytes, serum albumin, urea and creatinine were analysed in the Mulago National Referral hospital laboratories. The CBCs were analysed in the haematology laboratory using the SysmexXs 800i machine. The blood cultures were done in the Microbiology laboratory. The electrolytes were analysed in the clinical chemistry laboratory using a Cobas R 6000c501clinical chemistry analyser (Roche Diagnostics, Indianapolis, IN). All the results were recorded in the questionnaires. A structured questionnaire was used to collect information about the patients. It was pretested on 5 patients before the actual data collection to determine its applicability and suitability. The pretesting was done by one of us (AT). The study instrument was used to collect data on the socio-demographic characteristics, patient’s history, physical examination, laboratory results and to record the final outcome of the study participants.

#### Patient management and feedback

Results of the patients were promptly availed to the clinical team on the ward. Where an urgent intervention was required, the study team initiated management in consultation with the attending clinicians. Currently, there are no guidelines on the management of hyperglycaemia in non-diabetic children because the burden and clinical significance of the problem among children with severe malnutrition in our setting is not known due to limited studies in the field. Thus, for children who had hyperglycaemia, the results were communicated to the attending team for follow up.

#### Outcome/dependent variables

The primary outcome variables included a random blood sugar more than 150 mg/dl and death. Independent variables include patient’s general clinical condition, type of feed given (F75/SDTM), co-morbid conditions such as fever, convulsions, septicaemia, HIV serology of the patient and treatment received such as antibiotics and intravenous fluids (Dextrose).

#### Data management and analysis

Data was entered into EPI DATA version 3.1 and exported into STATA version 12. The data was checked for errors, cleaned and coded in preparation for analysis. Descriptive analysis was done using medians and inter-quartile ranges. Frequencies were also tabulated for other demographic and background characteristics. To answer objective 1, a new binary variable for hyperglycaemia was created. A patient was considered to have hyperglycaemia if he or she had a random blood sugar greater than 150 mg/dl.

To determine the factors associated with hyperglycaemia**,** bivariable analysis was done by cross tabulating the hyperglycaemia variable with the demographic characteristics, clinical symptoms of the children with severe malnutrition on admission to MNU, feeding history and co-morbid conditions of the children with SAM admitted to MNU and other factors. A simple chi square test was done at bivariate level and odds ratios and *p*-values were tabulated.

Factors with a *p*-value less than 0.05 were considered to be statistically significant.

For determination of the final outcome of the study participants:

A binary outcome variable was created (0 = no death, 1 = death). A cross tabulation was done for the binary outcome variable of death with that of stress hyperglycaemia, to get the frequencies of those with and without stress hyperglycaemia who died. Then bivariate analysis was done, to compare mortality between those with stress hyperglycaemia and those without.

#### Ethical considerations

Approval for the study was obtained from the Department of Paediatrics and Child Health of Makerere University; the School of Medicine Research and Ethics Committee (SOMREC) and the National Council for Science and Technology. Informed written consent was obtained from the care givers of the children.

## Results

### Description of the study population

**Fig. 1:**
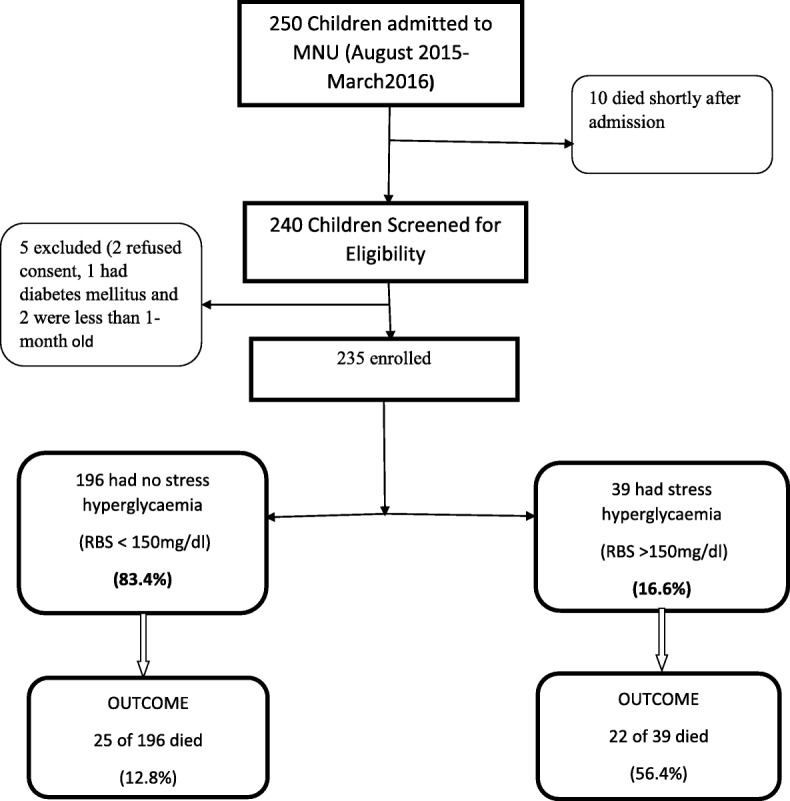
Study Profile. Between August 2015 and March 2016, a total of 250 children were admitted to the Mwanamugimu nutrition unit of Mulago hospital. Of these, 240 children were screened and 235 were found eligible and enrolled into the study. 16.6% of the children had stress hyperglycaemia. Of the children with stress hyperglycaemia, 56.4% died compared to 12.8% of the children who did not have stress hyperglycaemia

Between August 2015 and March 2016, a total of 250 children were admitted to the Mwanamugimu nutrition unit of Mulago hospital. Of these, 240 children were screened and 235 were found eligible and enrolled into the study. (Fig. 1).

### Baseline characteristics of the children with severe malnutrition admitted to Mwanamugimu nutrition unit

As seen in Table 1, 14 % of the participants were aged less than 6 months and 86% were above 6 months. The mean age was 15.1 months. Most of the children (87%) were cared for by their mothers; and 65% were from Kampala and the neighbouring Wakiso district (Table 1).

**Table 1:** Baseline characteristics of the children with severe malnutrition admitted to Mwanamugimu nutrition unit

	Frequency (*N* = 235)	Percentage
Age of patient (month)		
*< 6 months*	33	14
*≥ 6 months*	202	86
Sex of Patient		
*Boys*	132	56
*Girls*	103	44
Present district of residence		
*Kampala*	96	41
*Wakiso*	56	24
*Other*	83	35
Religion of the patient		
*Christians*	149	63
*Muslim*	86	37
Relationship of next of kin with the child		
*Mother*	203	87
*Other*^a^	32	13
Education level of the caretaker		
*none/primary*	140	60
*secondary/tertiary/post tertiary*	95	40
Occupation of the caretaker		
Professional/self employed	144	62
Housewife	89	38
Breast feeding		
Yes	51	22
No	183	78

^a^ *others included grandmothers, fathers and aunties*

### Clinical characteristics of the patients on admission

Table 2 shows the clinical presentation of the patients on admission. Fifteen percent of the children were lethargic and 9% required oxygen therapy. There were more children with non-oedematous malnutrition (51%) and the rest (49%) had oedematous malnutrition (Table 2).

**Table 2:** Clinical characteristics of the children with severe malnutrition on admission

	Frequency (235)	Percentage
General condition of the patient		
*Alert; n*	199	85
*Lethargic; n*	36	15
Oral sores; *n*	57	11
Hyperthermia (> 38.5°c); *n*	35	15
Pallor; *n*	162	70
Dermatosis; *n*	103	44
Respiratory distress; *n*	29	12
Patient on oxygen; *n*	21	9
Deep acidotic breathing; *n*	8	3
Malnutrition classification		
*SAM Non-oedematous; n*	121	51
*SAM Oedematous; n*	114	49

### Prevalence of stress hyperglycaemia among the participants

Of the 235 children aged 1–60 months with severe acute malnutrition enrolled into the study, 39 (16.6%) had hyperglycaemia (RBS > 150 mg/dl). The mean RBS was 115.5 mg/dl (SD 43.08) for all the participants. and 1.3% of the participants had hypoglycaemia (RBS < 60 mg/dl), 82.1% had RBS within the acceptable.

Prevalence of stress hyperglycaemia among the different clinical symptoms of the children with severe malnutrition admitted to MNU is shown in Table 3 (Table 3).

**Table 3:** Bivariate analysis of the clinical symptoms of the study participants (*N* = 235)

Clinical Characteristics	Stress hyperglycemia (*n* = 39)	No stress hyperglycemia (*n* = 196)	Odds ratio (95% CI)	*p*-value
Convulsion; *n* (%)	4 (10)	6 (3)	3.62 (1.0, 13.5)	0.042
Respiratory distress; *n* (%)	12 (31)	26 (13)	2.91 (1.3, 6.4)	0.007
Diarrhea; *n* (%)	30 (77)	106 (54)	2.90 (1.3, 6.3)	0.008
Oral sores; *n* (%)	18 (46)	39 (20)	3.45 (1.7, 7.1)	0.001
Dermatosis; *n* (%)	23 (59)	80 (41)	1.62 (1.2, 4.2)	0.039
Patient on oxygen; *n* (%)	7 (18)	14 (7)	2.84 (1.1, 7.6)	0.031
Malnutrition classification				
*SAM Non-edematous; n* (%)	18 (46)	103 (53)	1	
*SAM Edematous; n* (%)	21 (54)	93 (47)	1.29 (0.6, 2.6)	0.465
Duration of 10% dextrose before blood drawn				
*> 12 h but < 24 h n* (%)	16 (70)	12 (92)	1	
*> 24 h; n* (%)	7 (30)	1 (8)	0.19 (0.02, 1.7)	0.144

### Prevalence of stress hyperglycaemia among the different clinical symptoms of the children with severe malnutrition admitted to MNU

Table 3 shows the prevalence of stress hyperglycaemia among the participants with different clinical symptoms.

### Bivariate and multivariate analysis for factors independently associated with stress hyperglycaemia

On multivariate analysis (Table 4), only presence of oral sores was significantly associated with stress hyperglycaemia OR 2.611(95% CI 1.02, 6.65), *p*-value 0.044).

**Table 4:** Bivariate and multivariate analysis for the factors associated with stress hyperglycaemia

Variable	Stress hyperglycemia (*n* = 39)	No stress hyperglycemia (*n* = 196)	Crude (Unadjusted) Odds Ratio (95% Confidence Interval)	Adjusted Odds Ratio (95% Confidence Interval)	*p*-value
Age of patient (months)					
< 6 months	1	32	1	1	
≥ 6 months	38	156	4.74 (1.10–20.25)	2.274 (0.31–16.47)	0.416
Convulsion (*n*)	4	6	3.62 (1.0–13.5)	2.984 (0.53–16.72)	0.214
Respiratory distress (*n*)	12	26	2.91 (1.3–6.4(	1.798 (0.24–13.44)	0.568
Oral sores (*n*)	18	39	3.45 (1.7–7.1)0	2.611 (1.02–6.65)	0.044
Dermatosis (*n*)	23	80	1.62 (1.2–4.2)	1.132 (0.45–2.85)	0.793
Severe Pneumonia (*n*)	7	14	1.450 (0.72–2.89)	1.128 (0.50–2.54)	0.067
Type of feed					
F-75	38	162	1	1	
SDTM	1	34	0.13 (0.02–0.9)	0.124 (0.01–1.60)	0.110
Received 10% Dextrose (*n*)	13	24	3.58 (1.6–8.0)	1.474 (0.512–4.25)	0.473
Diarrhea					
Persistent diarrhea (*n*)	14	24	4.71 (1.90–11.71)	2.393 (0.80–7.17)	0.120
Acute diarrhea (*n*)	14	81	1.390	0.702 (0.24–2.04)	0.517

### Outcome of children with stress hyperglycaemia admitted to MNU

The overall mortality among the enrolled children was 20.2% (47 of 233). Among the 39 children with stress hyperglycaemia, 22 (56.4%) of the children died; while among the 194 children without stress hyperglycaemia, 25 (12.8%) of the children died.

Children with stress hyperglycaemia were 8 times more likely to die compared to the children without stress hyperglycaemia (OR 8.748, 95% CI 4.09–18.7).

## Discussion

We determined the prevalence, outcome and factors associated with stress hyperglycaemia among severely malnourished children in Uganda’s National referral hospital.

The prevalence of stress hyperglycaemia among children with severe acute malnutrition was 16.6%; meaning that almost 2 in every 10 children with severe acute malnutrition had stress hyperglycaemia. This is comparable to what has been found elsewhere [9, 19]. There is paucity of data on stress hyperglycaemia among children with severe malnutrition in low income countries. Bareness et al. in 2016 reported a prevalence of hyperglycaemia of 19.5% among children with severe acute malnutrition admitted to a referral hospital in Laos in Asia [9]. The slightly higher prevalence in that study could have been due to the small number of children with severe malnutrition because out of 350 children enrolled, only 46 had severe malnutrition. Our study assessed more children with severe malnutrition.

Faustino et al. in 2005 found a similar prevalence of stress hyperglycaemia of 16.7%.That study evaluated critically ill children in a paediatric intensive care unit without specifically looking at the severely malnourished [19].

The prevalence of stress hyperglycaemia in the current study is higher than what Sambany et al. found in Madagascar. They found a prevalence of stress hyperglycaemia of 10.5% among children with severe acute malnutrition [14]. These authors however, looked at all children admitted to a paediatric ward of a referral hospital, 74% of whom had severe malnutrition [14]. Various studies have shown that stress hyperglycaemia occurs among critically ill children [9, 12, 20, 21]. Of note, however, is the paucity of studies on stress hyperglycaemia among children with severe malnutrition.

Malnutrition is usually associated with various complications which put the body through a stressful period and hence the likelihood of developing stress hyperglycaemia. This is of great concern especially since the current WHO guidelines for management of children with SAM in resource limited settings recommend 10% dextrose on admission [6, 7]. This is based on the presumption that children with SAM are hypoglycaemic. Due to limited resources, we are not able to routinely check the random blood sugar levels for all the children with severe acute malnutrition on admission.

Often, clinicians do not adequately assess the risk of hypoglycaemia and opt to give all the children with SAM a bolus of 10% dextrose on admission. This might cause deterioration of hyperglycaemic children.

### Factors associated with stress hyperglycaemia among children with severe malnutrition

In the current study, a number of factors were associated with hyperglycaemia at bivariate analysis and these included convulsions, cough, diarrhoea, oral sores, and respiratory distress, being on oxygen, dermatoses and lethargy. Most of the factors that were significant at bivariate analysis overlap and are associated with increased severity of illness. Stress hyperglycaemia has been showed to occur more commonly in critically ill children [21].

Age ≥ 6 months was also significantly associated with stress hyperglycaemia at bivariate analysis. This could be attributed to the fact that children aged more than 6 months receive a different formulation of feeds (F75) which has more calories (75 kcal/100 ml) than the SDTM given to those below 6 months which contains 69 kcal/100 ml [6]. Children above 6 months of age also tend to be more critically ill.

### Stress hyperglycaemia among children with oral sores

Children who had oral sores were almost 3 times more likely to have stress hyperglycaemia. None of the previous studies had shown this direct relationship. However, basing on the mechanism of development of stress hyperglycaemia, this is in keeping with the pathophysiology from the literature. Severe acute malnutrition causes a defect in the immunity of the children thus making them susceptible to a number of infections [22, 23]. Some of these infections include viruses such as herpes simplex which commonly causes oral sores. Presence of infections leads to inflammatory response and subsequent stress hyperglycaemia [14]. Vitamin A and Zinc deficiencies which usually occur in children with severe malnutrition can cause oral sores [24, 25]. Children with micronutrient deficiencies also have compromised immunity which makes them susceptible to stress hyperglycaemia. Children who present with oral sores are also likely to be critically ill with other complications thus making them more likely to have stress hyperglycaemia [21].

### Outcome of children with stress hyperglycaemia

We found a very high mortality (56.4%) among children with stress hyperglycaemia compared to those without stress hyperglycaemia who had a mortality of 12.8%. These findings are similar to what has been found elsewhere. For example; Khan et al. in 2015 found a mortality of 57% among critically ill children with stress hyperglycaemia admitted in the Paediatric Intensive Care Unit, while in those with normal blood glucose it was 35% [23].

Vinayak et al. also found a higher mortality of 28.6% among children with hyperglycaemia compared to 3.2% among those without among children in an emergency unit [14]. Li Y et al. found that children with stress hyperglycaemia were 4 times more likely to die compared to the other children who had normal blood glucose levels. There is not enough data on the exact mechanism of hyperglycaemia leading to mortality but it has been found to be an important predictor of mortality in critically ill children.

### Limitations of the study

The current study had some limitations; we did not do specific tests to screen for possible diabetes mellitus among the participants such as urine for ketones and HbA1c. This limitation is unlikely to have affected the final results given that previous studies have demonstrated no association between stress hyperglycaemia and later development of diabetes mellitus among children. A urine dipstick is sometimes used to check for presence of ketones to differentiate between stress hyperglycaemia and diabetic ketoacidosis. This however, is not specific in children with severe malnutrition who are likely to have urine ketones secondary to increased gluconeogenesis from chronic starvation. Recall bias is also a possibility since most of the information like the feeding history was obtained from interviewing care givers of the children. These results may not be representative of the general population since the sampling was only done in one hospital due to limited resources.

## Conclusions

Stress hyperglycaemia is a common problem among children with severe acute malnutrition occurring in almost 2 in 10 children admitted to Mwanamugimu nutrition unit of Mulago Hospital in Uganda. Children with severe acute malnutrition who are critically ill with oral sores are more likely to have stress hyperglycaemia. Stress hyperglycaemia is associated with very high mortality among children with severe acute malnutrition admitted to the nutrition ward. It is important to check the random blood sugar level of critically ill children with severe acute malnutrition before giving any boluses of 10% dextrose. Innovative ways of preventing and managing stress hyperglycaemia are urgently needed.

## References

[1] UNICEF. WHO-The World Bank. 2014b" Summary of key facts about the 2013 joint malnutrition estimates.".

[2] Bandsma RH, Spoelstra MN, Mari A, Mendel M, van Rheenen PF, Senga E (2011). Impaired glucose absorption in children with severe malnutrition. J Pediatr.

[3] Bandsma RH, Mendel M, Spoelstra MN, Reijngoud DJ, Boer T, Stellaard F (2010). Mechanisms behind decreased endogenous glucose production in malnourished children. Pediatr Res.

[4] Spoelstra MN, Mari A, Mendel M, Senga E, van Rheenen P, van Dijk TH (2012). Kwashiorkor and marasmus are both associated with impaired glucose clearance related to pancreatic beta-cell dysfunction. Metab Clin Exp.

[5] Bhutia TD, Lodha R, Kabra SK (2013). Abnormalities in glucose homeostasis in critically ill children. Pediatr Crit Care Med.

[6] Uganda MoH. Guidelines for intergrated management of acute malnutrition in Uganda. In: Health, editor. 2016.

[7] Organization WH. Guideline: updates on the management of severe acute malnutrition in infants and children: World Health Organization (WHO); 2013. 24649519

[8] Mbabazi N, Mworozi E, Achan J, E M. Prevalence, presentation and immediate outcome of critically ill children with hypoglycemia presenting to the Acute Care Unit of Mulago hospital: Makerere University; 2011.

[9] Barennes H, Sayavong E, Pussard E (2016). High mortality risk in hypoglycemic and Dysglycemic children admitted at a referral Hospital in a non Malaria Tropical Setting of a low income country. PLoS One.

[10] Godinjak A, Iglica A, Burekovic A, Jusufovic S, Ajanovic A, Tancica I (2015). Hyperglycemia in Critically Ill Patients: Management and Prognosis. Medical archives (Sarajevo, Bosnia and Herzegovina).

[11] Preissig CM, Hansen I, Roerig PL, Rigby MR (2008). A protocolized approach to identify and manage hyperglycemia in a pediatric critical care unit. Pediatr Crit Care Med.

[12] Roberts GW, Quinn SJ, Valentine N, Alhawassi T, O'Dea H, Stranks SN (2015). Relative hyperglycemia, a marker of critical illness: introducing the stress hyperglycemia ratio. J Clin Endocrinol Metab.

[13] Srinivasan V (2012). Stress hyperglycemia in pediatric critical illness: the intensive care unit adds to the stress!. J Diabetes Sci Technol.

[14] Sambany E, Pussard E, Rajaonarivo C, Raobijaona H, Barennes H. Childhood dysglycemia: prevalence and outcome in a referral hospital. PLoS Med. 2013; 10.1372(0065193). 10.1371/journal.pone.0065193 PMC3669285 23741481

[15] Wintergerst KA, Buckingham B, Gandrud L, Wong BJ, Kache S, Wilson DM (2006). Association of hypoglycemia, hyperglycemia, and glucose variability with morbidity and death in the pediatric intensive care unit. Pediatrics.

[16] Valerio G, Franzese A, Carlin E, Pecile P, Perini R, Tenore A (2001). High prevalence of stress hyperglycaemia in children with febrile seizures and traumatic injuries. Acta Paediatr.

[17] Weiss SL, Alexander J, Agus MS (2010). Extreme stress hyperglycemia during acute illness in a pediatric emergency department. Pediatr Emerg Care.

[18] Kish L. Sampling organizations and groups of unequal sizes. Am Sociol Rev. 1965:564–72. 14325826

[19] Faustino EV, Apkon M (2005). Persistent hyperglycemia in critically ill children. J Pediatr.

[20] Digman C, Borto D, Nasraway SA (2005). Hyperglycemia in the critically ill. Nutr Clin Care.

[21] Patki VK, Chougule SB (2014). Hyperglycemia in critically ill children. Indian J Crit Care Med.

[22] Beck MA, Handy J, Levander OA (2004). Host nutritional status: the neglected virulence factor. Trends Microbiol.

[23] Jones KD, Berkley JA (2014). Severe acute malnutrition and infection. Paediatr Int Child Health.

[24] Wiseman EM, Bar-El Dadon S, Reifen R. The vicious cycle of vitamin a deficiency: a review. Crit Rev Food Sci Nutr. 2016;57(17)3703–14. 10.1080/10408398.2016.1160362 27128154

[25] Ackland ML, Michalczyk AA. Zinc and infant nutrition. Arch Biochem Biophys. 2016;611:51–57. 10.1016/j.abb.2016.06.011 27317042

